# Dynamics of *Apis mellifera* Filamentous Virus (AmFV) Infections in Honey Bees and Relationships with Other Parasites

**DOI:** 10.3390/v7052654

**Published:** 2015-05-22

**Authors:** Ulrike Hartmann, Eva Forsgren, Jean-Daniel Charrière, Peter Neumann, Laurent Gauthier

**Affiliations:** 1 Agroscope, Swiss Bee Research Center, Schwarzenburgstrasse 161, Bern 3003, Switzerland; E-Mails: uli.hartmann@gmx.ch (U.H.); jean-daniel.charriere@agroscope.admin.ch (J.-D.C.); peter.neumann@vetsuisse.unibe.ch (P.N.); 2 Department of Ecology, Swedish University of Agricultural Sciences, Uppsala 750 07, Sweden; E-Mail: Eva.Forsgren@slu.se; 3 Institute of Bee Health, Vetsuisse Faculty, University of Bern, Bern 3001, Switzerland

**Keywords:** *Apis mellifera*, filamentous virus, microsporidia, trypanosome, RNA viruses

## Abstract

*Apis mellifera* filamentous virus (AmFV) is a large double stranded DNA virus of honey bees, but its relationship with other parasites and prevalence are poorly known. We analyzed individual honey bees from three colonies at different times post emergence in order to monitor the dynamics of the AmFV gut colonization under natural conditions. Prevalence and loads of microsporidia and trypanosomes were also recorded, as well as five common honey bee RNA viruses. The results show that a high proportion of bees get infected with AmFV during the first week post-emergence (75%) and that AmFV DNA levels remained constant. A similar pattern was observed for microsporidia while trypanosomes seem to require more time to colonize the gut. No significant associations between these three infections were found, but significant positive correlations were observed between AmFV and RNA viruses. In parallel, the prevalence of AmFV in France and Sweden was assessed from pooled honey bee workers. The data indicate that AmFV is almost ubiquitous, and does not seem to follow seasonal patterns, although higher viral loads were significantly detected in spring. A high prevalence of AmFV was also found in winter bees, without obvious impact on overwintering of the colonies.

## 1. Introduction

Besides their economic importance as major crop pollinators, honey bees are tight members of natural ecosystems in which they play fundamental roles [1]. However, because they overlap with human activities, pollinators are currently threatened by either/or biotic and abiotic factors [2,3,4]. In particular, they have to suffer the introduction of invasive species resulting from commercial exchanges, such as *Varroa destructor* [5] or *Aethina thumida* [6], alongside the extensive use of agrochemicals [7]. In parallel, plant biodiversity has been considerably reduced in many areas resulting in temporary limitations in food resources. All these factors may concur to weaken the bee colony immune defense and to enhance the development and spread of opportunistic infections [8,9,10,11,12,13].

The relationships between microorganisms within the bee colony are tightly balanced by their environment and some of these may become pathogenic for honey bees under stressing conditions or when triggering factors are present, leading ultimately to the production of clinical signs at the colony level. This has been demonstrated for some facultative pathogens such as *Serratia sp*. which can be involved in septicemia in insects [14] and for some honey bee viruses which normally produce covert infections, but can become acute infections when colonies are highly infested by the mite *V. destructor* [15]. Most of the viruses identified in honey bees to date are small RNA viruses and only a single DNA virus, the *A. mellifera* filamentous virus (AmFV), has been described [16] and sequenced [17]. The viral double stranded DNA genome is encapsidated in a long nucleocapsid of 3000 × 40 nm length and coiled within a membrane [18,19]. A first molecular diagnosis of AmFV virus has shown that it is prevalent in honey bee colonies and presumably transmitted both horizontally by food exchanges and vertically from the queen to worker progeny [17]. The virus has also been detected in solitary bee species suggesting that AmFV has a large host spectrum [20]. Acute infections of honey bees by AmFV lead to the lysis of bee tissues such as fat body, resulting in the presence of characteristic signs in which the worker hemolymph appear milky-white; these infected bees are often found crawling at the hive entrance [21]. However, symptoms linked to AmFV acute infections are rare and have barely been associated with colony losses in the past [16,18], despite the presence of putative functions involved in virulence in the AmFV genome [17]. Therefore, in light of the current and repeated colony losses reported by beekeepers, it is still questionable whether AmFV could be pathogenic, especially in the context of multi-infections with other parasites such as the microsporidia *Nosema apis* and *Nosema ceranae* [22,23], the trypanosomes *Crithidia mellificae* and *Lotmaria passim* [24,25] or RNA viruses [15]. Indeed it has been previously suggested that AmFV replication could be associated with Black queen cell virus (BQCV) and with *N. apis* infections [26,27]. BQCV is a very common RNA virus which produces covert infections in honey bees [28]. *N. apis* was for a long time the only species known from western honey bees [29] but nowadays this parasite seems more scarcely detected in certain areas and replaced by another species, *N. ceranae*, which is highly prevalent in Europe and in the USA [22,30,31]. Although *N. apis* occasionally produce acute infections in the gut leading to diarrhea [27], no clear clinical signs have yet been associated with *N. ceranae* in natural conditions [32]. However, part of the current literature considers this species as a devastating pathogen that might be involved in the CCD (Colony Collapse Disorder) syndrome in the USA [33,34]. In contrast, no clear connections between *N. ceranae* infections and colony death could be established in Europe [34,35,36], with the exception of Spain [37].

Regarding the environmental changes during the last two decades, in particular following the global spread of *V. destructor* and the identification of *N. ceranae* in large areas, we aimed at defining the relationship between AmFV and several other common honey bee parasites under natural conditions. This study also includes the flagellates *C. mellificae* and *L. passim* which have recently been characterized on a molecular level and seem to be ubiquitous in US apiaries [25,38]. As the bee midgut is an initial target for most of the bee parasites [39], we first attempted to monitor the kinetics of gut invasion by these parasites in individual bees.

## 2. Materials and Methods

### 2.1. Honey Bee Samples

In July 2010, sealed worker brood combs were collected from three healthy colonies (A, B and C) located in Bern (Switzerland) and placed overnight in an incubator at 34.5 °C. Emerging workers were marked on the thorax and returned to their original hive. Age cohorts of eight workers were collected in each colony one, two and three weeks after emergence and the guts were immediately removed by carefully pulling the last segment of the abdomen using a sterile forceps. Guts collected from bees at emergence were used as controls. For winter bee analyses, eight adult workers were collected from each of these three colonies in January 2011. These three colonies overwintered successfully and were still healthy in spring 2011.

Adult bee samples collected in 2002 from 50 colonies in France (5 apiaries, 10 colonies in each) [40] were used for assessing the prevalence of AmFV at the colony level. Additionally, a set of DNA samples previously extracted from pooled bees and originating from 86 colonies (37 apiaries) in Sweden were used because of the presence of *N. apis* in these samples. The Swedish samples were collected in 2009 and part of a survey published in 2013 [41].

### 2.2. DNA Extraction and PCR Analysis

The bee digestive tract was homogenized in 250 µL of a denaturing Buffer (Buffer T1, Nucleospin Tissue^®^ kit, Macherey-Nagel) using a 5 mm metal bead and the Tissue Lyzer^®^ homogenizer (Qiagen) for 30 s, at maximal frequency. Fifty microliters of the resulting homogenate was used for DNA extraction, using the NucleoSpin^®^ Tissue kit (Macherey Nagel) following the manufacturer’s recommendations. The DNA was quantified by spectrophotometry (Nanodrop^®^) after elution in 100 µL of water and used for PCR analysis. The DNA extraction step was ascertained by detecting the actin gene in the samples using the qPCR technique described below. In parallel, crude extracts of bees collected in 2002 from 50 colonies and kept at −20 °C were used for PCR detection of parasites following the protocol described above. Additionally, a set of DNA samples previously extracted from pooled bees and originating from 86 colonies in Sweden were used because of the presence of *N. apis* in these samples.

Qualitative PCR analyses for AmFV, *N. ceranae*, *N. apis* and *C. mellificae/L. passim* were performed in a 50 µL final volume using the Goldstar^®^ DNA Polymerase (Eurogentec). A list of primers is presented in supplementary files (Supplement Table S1). PCR conditions were as follows: 2 min at 94 °C and 30 cycles of 30 sec at 94 °C, 30 sec at 56 °C and 30, 45 or 60 sec at 72 °C for *N. ceranae* and *N. apis*, AmFV, *C. mellificae/L. passim*, respectively, followed by a final elongation step of 7 min at 72 °C.

Quantitative PCR analyses were performed using the SYBR^®^ Green I Mastermix kit (Eurogentec) in 12 µL final volume and using a RotorGene-3000A (Corbett Research). PCR conditions were 50 °C for 2 min, 95 °C for 10 min and 40 cycles at 95 °C for 15 sec and 60 °C for 1 min. Each sample was analyzed in duplicate and positive and non-template controls were included in each run. For AmFV quantification, primers were designed from a baculovirus-like sequence encompassing a putative BroN domain in the AmFV genome [17] using the Primer Express^®^ software (Applied Biosystems). Primers are presented in supplementary files (Supplement Table S1), together with qPCR performance indicators. The qPCR data were converted to the initial amount of each target in the reaction (N_0_) using the LinReg software [42]. The AmFV titers were deduced from N_0_ values calculated from standard curves made of serial dilutions of known amounts of the amplicons. Quantitative PCR for *N. ceranae* was performed following a published protocol [43]. Data were normalized according to the *A. mellifera* β-actin reference gene [44] and are presented as equivalent AmFV genome copies.

### 2.3. Statistical Analyses

Statistics were performed with the programs Systat^®^ and R. Since the data failed tests for normality, the following tests were used: Chi^2^-test, Mann-Whitney U test, Kruskal-Wallis test, binary logistic regression, general linear model, robust linear model and Spearman rank correlation.

## 3. Results

### 3.1. Kinetics of Honey Bee Gut Colonization by Parasites

Honey bee workers were collected at one week intervals from three healthy colonies and DNA was separately extracted from guts and bodies prior to PCR analysis. As shown in Table 1, no AmFV, *N. ceranae*, *N. apis* and *C. mellificae/L. passim* were detected in guts collected from emerging bees. These pathogens were also not detected in the rest of the body of emerging workers (Table 2). Conversely, a majority of older workers were found infected with AmFV, *N. ceranae* and *C. mellificae/L. passim*. AmFV was detected in 75%, 65% and 63% of the guts, and in 100%, 79%, 87% of the bodies, in the 1st, 2nd and 3rd week after emergence, respectively (Table 1 and Table 2). The virus was also detected in gut and bodies of winter bees (96% and 71%, respectively). In the guts, the prevalence for *N. ceranae* increased from 17% in the 1st week to 30% in the second week and to 71% in the 3rd week post emergence (Table 1). In the bodies, *N. ceranae* prevalence was higher (75%, 37% and 100% in the 1st, 2nd and 3rd week post emergence, respectively) (Table 2). The percentage of winter bees infected with this microsporidia was 83% in guts and 87% in bodies. No *C. mellificae/L. passim* were detected in the first week after emergence while 30% and 42% of the guts were found infected in the 2nd and 3rd week, respectively. In the bodies, 12%, 33% and 58% were found positive for trypanosome in the 1st, 2nd and 3rd week post emergence, respectively. In winter bees, a higher prevalence for *C. mellificae/L. passim* was recorded in guts than in bodies (54% and 17%, respectively). No *N. apis* was detected in any of these samples. AmFV loads in these bees were estimated by quantitative PCR and showed no significant differences according to the date post emergence (*p* > 0.05). AmFV loads were estimated around 1 × 10^5^ equivalent genome copies per honey bee gut in average.

**Table 1:** Detection of *Apis mellifera* Filamentous virus (AmFV), *Nosema ceranae* and *C. mellificae/L. passim* from individual bees at 0, 1, 2 or 3 weeks after emergence (weeks p.e.) and in winter bees (WB) (*n* = 24 workers from 3 colonies). +: Percentage of PCR positive intestines. *n*: Samples not analyzed due to failed DNA extraction.

GUTS	AmFV					*N. ceranae*					Trypanosomes				
Weeks p.e.	0	1	2	3	WB	0	1	2	3	WB	0	1	2	3	WB
Colony A		+		+	+				+	+					
		+		+	+					+					
		+	*n*	+	+			*n*	+	+			*n*		
			+		+			+	+	+			+	+	
		+	+	+	+		+		+	+			+	+	
		+		+	+					+				+	+
			+	+	+			+		+				+	
		+	+	+	+		+		+	+					+
Colony B		+			+			+	+				+	+	+
			+		+			+	+	+				+	
		+	+		+		+		+	+					+
		+	+		+		+		+					+	
		+	+		+				+	+				+	+
		+		+	+				+						+
		+			+				+	+			+		
		+			+					+				+	+
Colony C			+	+	+			+	+	+			+		
		+		+	+			+		+					+
			+	+	+								+		
		+	+	+	+			+	+	+			+	+	+
		+	+	+	+					+					+
		+	+	+					+	+					+
			+	+	+				+	+					+
		+	+		+				+	+					+
pos. (%)	0	75	65	63	96	0	17	30	71	83	0	0	30	42	54

**Table 2:** Detection of *Apis mellifera* Filamentous virus (AmFV), *Nosema ceranae* and *C. mellificae/L. passim* in bodies (after gut removal) from individual bees at 0, 1, 2 or 3 weeks after emergence (weeks p.e.) and in winter bees (WB) (*n* = 24 workers from 3 colonies). +: Percentage of PCR positive bodies.

BODIES	AmFV					*N. ceranae*					Trypanosomes				
Weeks p.e.	0	1	2	3	WB	0	1	2	3	WB	0	1	2	3	WB
Colony A		+							+	+			+		
		+		+	+		+		+	+					
		+	+	+	+			+	+	+					
		+	+					+	+	+			+		
		+	+	+				+	+				+		
		+		+	+		+		+	+		+			
		+	+	+					+	+				+	
		+	+	+	+		+		+	+				+	
Colony B		+		+	+		+		+	+			+		
		+		+	+		+	+	+	+			+	+	+
		+	+	+	+		+		+	+					
		+	+	+	+		+	+	+	+					
		+	+	+			+	+	+	+					
		+	+	+	+		+		+	+				+	+
		+	+	+	+		+		+	+			+	+	
		+	+		+		+		+				+	+	+
Colony C		+	+	+	+		+	+	+	+				+	
		+	+	+	+		+		+	+			+	+	
		+	+	+	+		+	+	+	+				+	
		+	+	+	+		+		+	+		+		+	+
		+	+	+	+		+	+	+	+				+	
		+	+	+	+		+		+	+				+	
		+	+	+			+		+	+		+		+	
		+	+	+					+					+	
pos. (%)	0	100	79	87	71	0	75	37	100	87	0	12	33	58	17

In the guts, PCR analysis showed that colony B was significantly less infected by AmFV than colony C (binary logistic regression: *p* < 0.01). No differences were observed between A and B or A *versus* C colonies respectively (*p* > 0.05). Similarly, no correlation was detected between AmFV and *C. mellificae/L. passim* or *N. ceranae* prevalence (*p* > 0.05). However, quantitative AmFV analysis showed differences between colonies. Colony A had significantly higher AmFV loads compared to colony B (general linear model: Tukey’s-Honestly-significant-difference test *p* < 0.01). No differences were detected between colony A and C or C and B (*p* > 0.05). Likewise, this model showed no association between AmFV loads and variables such as time after emergence, *C. mellificae/L. passim* or *N. ceranae* loads (*p* > 0.05). In winter bees, no significant associations were detected neither between AmFV and *N. ceranae* nor *C. mellificae/L. passim* prevalence (binary logistic regression *p* > 0.05). Data showed that *C. mellificae/L. passim* positive samples have significantly higher *N. ceranae* loads (General linear model; *p* < 0.05), but no significant connections between *N. ceranae* loads and variables such as weeks after emergence, colony and AmFV were detected (*p* > 0.05). A binary logistic regression showed no significant associations between *C. mellificae/L. passim* and variables such as colony, AmFV and *N. ceranae* loads (*p* > 0.05).

### 3.2. Relationships between AmFV and Honey Bee Parasites at the Colony Level

A set of pooled adult bee samples (100 workers) collected from 50 healthy honey bee colonies were used to estimate potential associations between AmFV and five common honey bee RNA viruses, namely Deformed wing virus (DWV), Sacbrood virus (SBV), Black queen cell virus (BQCV), Acute bee paralysis virus (ABPV) and Chronic bee paralysis virus (CBPV). These colonies were part of a large survey performed in France from pooled bee samples collected in 2002 from 36 apiaries in which virus loads were quantified using qPCR [45]. In these colonies, AmFV was detected in comparable percentages in spring (48%), summer (36%) and autumn (43%). *C. mellificae/L. passim* was detected in spring (56%), in summer (70%) and in autumn (63%). *N. ceranae* occurred in spring (44%), in summer (42%) and in autumn (49%) (see supplementary file Table S2). *N. apis* was only detected in spring in two colonies in the same apiary. No seasonal variation in AmFV prevalence was observed (Chi^2^-test, *p* > 0.05, Goodman-Kruskal’s Gamma G = 0.09). However AmFV loads were significantly higher in spring than in autumn (Kruskal-Wallis test: *p* < 0.05, H = 7.69 and Conover-Inman-test *p* < 0.01). No significant variations were detected between the other seasons (spring and summer: *p* > 0.05 and summer and fall: *p* > 0.05). A significant positive relationship was detected between prevalence of AmFV and *C. mellificae* in these colonies (binary logistic regression: *p* < 0.05), but AmFV loads were significantly higher in *C. mellificae* negative samples (robust linear model, *p* < 0.05). No relationships could be observed between AmFV and *N. ceranae* prevalence.

In spring and summer, significant positive correlations between AmFV loads and DWV (Spearman rank correlation: Spring: *p* < 0.001, r = 0.45; summer: *p* < 0.001, r = 0.49), SBV (spring: *p* < 0.0001, r = 0.53; summer: *p* < 0.05, r = 0.35) and BQCV (spring: *p* < 0.05, r = 0.30; summer: *p* < 0.01, r = 0.34) loads were detected (Supplement Figure S1). No significant correlations were observed either in spring or in summer between AmFV loads and CBPV/ABPV (*p* > 0.05) loads. In autumn, only BQCV loads (*p* < 0.05, r = 0.37) were significantly positively correlated with AmFV loads (*p* > 0.05). In this survey, *N. ceranae* positive colonies had significantly higher SBV loads (*p* < 0.0001). A model (binary logistic regression) showed no significant association between *N. ceranae* prevalence and variables such as season, *C. mellificae* detection or AmFV, DWV, ABPV and BQCV viral loads (*p* > 0.05).

**Figure 1:**
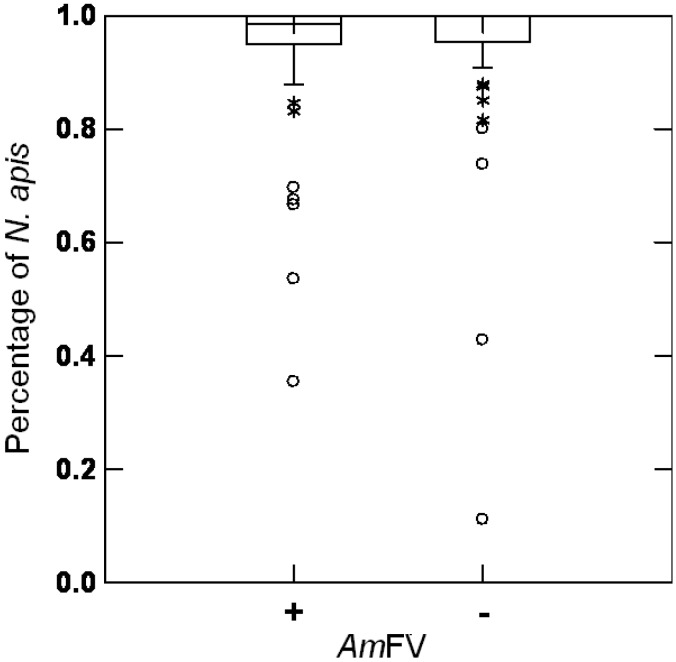
Proportion of *Nosema apis* positive colonies in *Apis mellifera* Filamentous virus (AmFV) positive and negative samples. Data were recorded from Swedish colonies (*n* = 86). No significant differences were detected.

Because *N. apis* was not detected in the Swiss and French colonies, the potential relationship between AmFV and *N. apis* was assessed using samples previously collected in Sweden (*n* = 86 colonies) [41]. The prevalence of AmFV was 53% in these colonies and all were positive for *N. apis* only, or infected with both *N. apis* and *N. ceranae*. The proportion of *N. apis* infected colonies was not significantly linked to AmFV infection (Mann-Whitney-U-test: *n* = 74, U = 3.13, *p* > 0.05) (Figure 1).

## 4. Discussion

### AmFV Distribution in Honey Bee Colonies

Previous data suggest that AmFV can be transmitted both horizontally and vertically [17]. The data presented here further show that AmFV can be highly prevalent among honey bee workers and that emerging bees get progressively infected during the first week of contact with their nest mates. During the first week post emergence the gut is also colonized by diverse symbionts, which will play a role in food digestion during the whole adult life [46,47]. The high number of AmFV positives bodies detected after one week post emergence was unexpected according that infections commonly initiate in the gut, and suggest a rapid dissemination of the virus in the hemocoel through the intestinal epithelium. However more data are needed to clearly understand the AmFV infectious process. During the following weeks, AmFV loads remained constant at moderate levels, thereby suggesting a chronic infection with low replication of the virus in the bee tissues once established. However our data cannot ascertain the replication of AmFV in the bee tissues, as high loads of the virus can also be detected in honey and pollen [17].

The seasonal prevalence of AmFV was investigated from pooled adult bee samples obtained from 50 healthy colonies from five apiaries, that were part of a larger survey of 36 apiaries located throughout France, sampled in spring, summer and autumn of 2002 [40,45]. Although all samples were found positive for AmFV, loads were significantly higher in spring, which is in line with previous observations using transmission electron microscopy for diagnosis [26]. The data therefore do not support an influence of *V. destructor* on AmFV prevalence and loads in honey bee colonies, since mite infestation rates are lower in spring than in summer and fall in Europe [5].

A synthesis of all data recorded from healthy and productive colonies, suggest that AmFV is not pathogenic for honey bees at the colony level. Therefore, the huge replication of AmFV associated with production of milky hemolymph symptoms observed in rare cases [20] could presumably be linked with the presence of other triggering factor(s). Possible factors that can alter the balance between viruses and their hosts are microorganisms such as microsporidia or trypanosomatids. One can speculate that such infections may act synergistically with viruses and produce symptoms leading to honey bee colony weakness or even to colony collapse [48]. Synergistic effects can occur, for example when the physical barriers of the honey bee gut, such as the peritrophic membrane or the basal lamina of intestinal cells, are ruptured by one parasite which may allow the passage of other parasites to secondary infections sites such as hemocytes or fat body cells. This is the reason why we monitored in parallel the common parasites *N. ceranae* and *C. mellificae/L. passim,* as well as five common honey bee RNA viruses.

*AmFV and Nosema spp*. The microsporidian *N. ceranae* is frequently cited in the literature to be involved in colony collapse, although recent data suggest that this link seems restricted to Spain [35,36,37,49]. In our assay conducted in Switzerland during summer, a high prevalence of *N. ceranae* was found among individual young workers confirming earlier findings [50]. This result is in agreement with the recent data from the USA [38] and Switzerland [49,50] showing a high prevalence as well as high loads of *N. ceranae* in bee samples during July and August. These values where much reduced during winter in the US survey. In contrast, our sampling showed a high proportion of *N. ceranae* positive winter bees. The discrepancies between data may result from the repeated treatments of US colonies with Fumagillin from August until October in this experiment. Fumagillin, an antibiotic prohibited in Europe, has however recently been shown to have no impact on colony overwintering in Canada [51] suggesting a low impact of these infections on honey bee health. Our data also indicate that *N. ceranae* do not impair colony overwintering in Switzerland. No relationship between the presence or the loads of *N. ceranae* and the detection of AmFV could be observed either from the study performed in Switzerland from individual bees collected in 3 colonies or from the survey performed in France on 50 colonies. Additionally, no relationship between AmFV and *N. apis* could be detected from the analysis of the 86 Swedish colonies [41]. The apparent lack of association between *N. apis* and AmFV contrasts with the results obtained previously in England [26] and deserve further investigations.

*AmFV and C. mellificae / L. passim*: Trypanosomes are widely distributed among insect orders [52] and honey bee microsporidia was described long ago [53] but only recently characterized on a molecular level [24,25]. Infections with *C. mellificae/ L. passim* seems to cause little or no harm [53], although *C. mellificae/L. passim* was found to correlate with honey bee colony mortality during winter [54]. Likewise *C. bombi*, a trypanosome occurring in the bumble bee *Bombus terrestris*, delays worker ovarian development and oviposition [55], and negatively impacts the development of workers [56]. It has been shown that *C. bombi* only infects older bumble bees [57]. In our study, we could also detect flagellates but only in individual bees collected at 2 and 3 weeks after emergence suggesting different colonization dynamics than *N. ceranae* or AmFV. In the US, a recent survey showed a large distribution of these parasites among honey bee colonies with a higher incidence in January and a positive association with *N. ceranae* infections [38]. Our data confirmed that *C. mellificae*/*L. passim* positive guts of individual bees have significantly higher *N. ceranae* loads. In contrast, at the colony level, our data showed that trypanosome negative samples had significantly higher AmFV titers, reflecting a potential negative relationship between these two parasites.

*AmFV and other viruses*: Positive correlations were detected in spring and summer between AmFV and DWV, SBV and BQCV, despite the fact that these three RNA viruses display different biological patterns [45]. DWV is strongly associated with the development of *V. destructor* colony infestations [15] and is consequently much more prevalent in summer and fall while SBV infections peak mostly in spring and summer and seem independent from mite infestations [45]. BQCV produces persistent infections in honey bees, mostly in spring and summer, and was only in rare cases detected in *V. destructor* [58]. Even though a connection between AmFV and BQCV has already been reported [26], additional work should be undertaken to clarify the correlations observed between AmFV and these three RNA viruses.

## 5. Conclusions

The present data indicate that the honey bee intestinal tract is frequently parasitized by microorganisms in absence of clinical signs observable at the individual bee and/or at the colony level. In our experiment, which reflects natural conditions, the colonization of the gut by AmFV and *N. ceranae* occurs during the first week after emergence for the majority of workers, concurrent with the colonization of the gut microflora. In contrast, *C. mellificae/L. passim* seem to infect older bees as these parasites were detected only during the second and third week after emergence. They were also largely found in winter bees collected from colonies that survived winter, suggesting an apparent low impact on honey bee colony health [49].

## Supplementary

Table S1. List of the primers used in the different screenings.

Table S2. Screening of different parasites from pools of 100 bees or mites collected from healthy colonies in 50 colonies (5 apiaries) in spring (I), summer (II) and autumn (III) [40]. DWV: Deformed wing virus; SBV: Sacbrood virus; CBPV: Chronic bee paralysis virus; ABPV: Acute bee paralysis virus; BQCV: Black queen cell virus. V: *Varroa destructor* sample. *n*: Sample not available. Apiary number is indicated on the left.

Figure S1. Correlations between quantities of AmFV recorded in pooled bee samples collected from 50 healthy colonies and three common honey bee RNA viruses: Deformed wing virus (DWV), Sacbrood virus (SBV) and Black queen cell virus (BQCV). Data are presented according to three samplings performed in spring, summer and fall 2002 [40]. Viral loads are expressed as equivalent genome copies. (Spearman rank correlation)
