# Supplementary material for: Dynamics of Apis mellifera Filamentous Virus (AmFV) Infections in Honey Bees and Relationships with Other Parasites
Source: Viruses. 2015 May 22;7(5):2654–67. doi: 10.3390/v7052654 (PMC4452924; doi:10.3390/v7052654)
Supplement: Supplementary file 1 [file viruses-07-02654-s001.zip › Hartmann-Primers-TableS1.pdf]

SUPPLEMENTARY FILE / Table 1.

	Target	primers	sequence (5' - 3')	size (bp)	Molarity	E	Tm (°C)	References
Qualitative PCR	*Am*FV	AmFV-F<br>AmFV-R	CAGAGAATTCGGTTTTTGTGAGTG<br>CATGGTGGCCAAGTCTTGCT	551	0.4 µM	n.a.	56	[1]
	N. ceranae	Ncer-F<br>Ncer-R	CGGATAAAAGAGTCCGTTACC<br>TGAGCAGGGTTCTAGGGAT	250	0.4 µM	n.a.	56	[2]
	N. apis	Napis-F<br>Napis-R	CCATTGCCGGATAAGAGAGT<br>CCACCAAAAACTCCCAAGAG	269	0.4 µM	n.a.	56	[2]
	C. mellificae	TryB-R106<br>TryA-R40	GTTGACGGAATCAACCAAACAAAT<br>GCGTCAGAGGTGAAATTCTTAGACC	715	0.4 µM	n.a.	56	[3]
	**Target**	**primers**	**sequence (5' - 3')**	**size (bp)**	**Molarity**	**E**	**Tm* (°C)**	**References**
Quantitative PCR	*Am*FV	AmFV2-F<br>AmFV2-R	ACCCAACCTTTTGCGAAGCGTT<br>ATGGGGCGTCTCGGGTAACCA	97	0.3 µM	1.93	77.6	This publication
	N. ceranae	qNcer-F<br>qNcer-R	AAGAGTGAGACCTATCAGCTAGTTG<br>ATCTCTCATCCCAAGAGCATTGC	104	0.3 µM	1.90	76.7	[4]
	β-Actin mRNA	A.m. β-Actin-q92F<br>A.m. β -Actin-q157R	CGTTGTCCCGAGGCTCTTT<br>TGTCTCATGAATACCGCAAGCT	66	0.4 µM	1.97	79.6	[5]

*Tm of the PCR amplicon calculated from the dissociation curve

References:

1. Gauthier, L.; Cornman, S.; Hartmann, H.; Cousserans, F.; Evans, J.D.; de Miranda, J.R.; Neumann, P. The *Apis mellifera* filamentous virus genome; submitted.
2. Chen, Y.P.; Evans, J.D.; Murphy, C.; Gutell, R.; Zuker, M.; Gundensen-Rindal, D.; Pettis, J.S. Morphological, molecular, and phylogenetic characterization of *Nosema ceranae*, a microsporidian parasite isolated from the European honey bee, *Apis mellifera*. *J Eukaryot Microbiol*. **2009**, 56(2):142-7.
3. Cox-Foster, D.L.; Conlan, S.; Holmes, E.C.; Palacios, G.; Evans, J.D.; Moran, N.A.; Quan, P.L.; Briese, T.; Hornig, M.; Geiser, D.M.; Martinson, V.; VanEngelsdorp, D.; Kalkstein, A.L.; Drysdale, A.; Hui, J.; Zhai, J.H.; Cui, L.W.; Hutchison, S.K.; Simons, J.F.; Egholm, M.; Pettis, J.S.; Lipkin, W.I. A metagenomic survey of microbes in honey bee colony collapse disorder, *Science* 2007, *318*, 283-287.
4. Bourgeois, A.L.; Rinderer, T.E.; Beaman, L.D.; Danka, R.G. Genetic detection and quantification of *Nosema apis* and *N. ceranae* in the honey bee, *J. Invertebr. Pathol* 2010, *103*, 53-58.
5. Gauthier, L.; Ravallec, M.; Tournaire, M.; Cousserans, F.; Bergoin, M.; Dainat, B.; de Miranda, J.R. Viruses associated with ovarian degeneration in *Apis mellifera* L. queens. *PLoS One*. 2011 Jan 25;6(1):
