# Supplementary material for: Dynamics of Apis mellifera Filamentous Virus (AmFV) Infections in Honey Bees and Relationships with Other Parasites
Source: Viruses. 2015 May 22;7(5):2654–67. doi: 10.3390/v7052654 (PMC4452924; doi:10.3390/v7052654)
Supplement: Supplementary file 1 [file viruses-07-02654-s001.zip › Table_S2.pdf]

		DWV			SBV			BQCV			ABPV			CBPV			*N. ceranae*			*Trypanosoma*			AmFV			
		I	II	III	I	II	III	I	II	III	I	II	III	I	II	III	I	II	III	I	II	III	I	II	III	V
150	1		+	+					+											+			+	+	+	
	2	+	+	+					+											+		+	+	+	+	
	3		+	+					+														+	+	+	+
	4		+	+				+	+											+			+	+	+	
	5		+	n			n		+	n			n			n		+	n			n	+	+	n	+
	6		+	+				+	+											+			+	+	+	
	7	+	+	+				+	+											+		+	+	+	+	
	8	+	+	+					+											+			+	+	+	
	9		+	+					+														+	+	+	
	10		+	+				+	+										+	+	+		+	+	+	
380	1	+	+	+	+	+		+	+		+	+					+				+		+	+	+	n
	2	+	+	+	+	+	+	+	+			+					+	+	+		+		+	+	+	n
	3	+	+	+		+	+	+	+	+	+						+	+	+		+	+	+	+	+	n
	4	+	+	+			+	+	+								+		+	+	+	+	+	+	+	n
	5	+	+	+	+	+		+				+									+	+	+	+	+	n
	6	+	+	+	+			+	+			+					+	+	+		+		+	+	+	n
	7	+	+	+	+	+	+	+		+	+						+	+	+	+	+	+	+	+	+	n
	8	+	+	+					+			+						+		+	+		+	+	+	n
	9	+	+	+		+		+	+	+							+	+	+	+	+		+	+	+	n
	10	+	+	+	+			+	+			+					+	+	+		+	+	+	+	+	n
426	1		+																+	+	+	+	+	+	+	n
	2																+			+	+	+	+	+	+	
	3		+	+												+					+	+	+	+	+	
	4		+	+												+				+	+	+	+	+	+	
	5	+						+											+	+	+		+	+	+	
	6	+		+																+	+	+	+	+	+	
	7	+		+				+												+	+	+	+	+	+	
	8		+	+															+		+	+	+	+	+	
	9	+	+	+				+													+	+	+	+	+	
	10	+	+	+				+						+							+	+	+	+	+	
624	1	+	+	+	+	+			+			+							+	+	+	+	+	+	+	n
	2	+	+	+	+					+										+		+	+	+	+	n
	3	+	+	+	+	+		+	+	+							+	+		+		+	+	+	+	n
	4	+	+	+		+			+							+					+	+	+	+	+	n
	5	+	+	+		+		+	+			+					+		+	+		+	+	+	+	n
	6	+	+	+	+	+		+	+			+							+	+	+		+	+	+	n
	7	+	+	+		+		+		+									+			+	+	+	+	n
	8	+	+	+	+	+		+	+												+		+	+	+	n
	9	+	+	+	+	+		+											+	+	+	+	+	+	+	n
	10	+	+	+	+	+		+	+								+	+					+	+	+	n
878	1	+	+	+	+	+	+		+								+	+	+	+	+	+	+	+	+	

	2	+	+	+	+	+		+									+	+	+			+	+	+	+	+
	3	+	+	+	+			+	+								+	+	+	+	+	+	+	+	+	
	4	+	+	+	+	+		+	+								+	+	+	+	+	+	+	+	+	
	5	+	+	+	+	+		+	+								+	+					+	+	+	
	6	+	+	+	+	+			+								+	+	+	+	+		+	+	+	
	7	+	+	+	+	+	+	+	+								+	+	+		+	+	+	+	+	
	8	+	+	+	+	+		+	+								+	+	+		+		+	+	+	
	9	+	+	+	+	+		+	+								+	+	+		+		+	+	+	
	10	+	+	+	+	+	+										+	+	+				+	+	+	
(%)		*76*	*92*	*94*	*46*	*48*	*14*	*62*	*66*	*24*	*6*	*9*	*0*	*2*	*0*	*6*	*44*	*40*	*51*	*54*	*66*	*56*	*100*	*100*	*100*	*10*
