# Supplementary figures and images for: Dynamics of Apis mellifera Filamentous Virus (AmFV) Infections in Honey Bees and Relationships with Other Parasites
Source: Viruses. 2015 May 22;7(5):2654–67. doi: 10.3390/v7052654 (PMC4452924; doi:10.3390/v7052654)

**Spring**

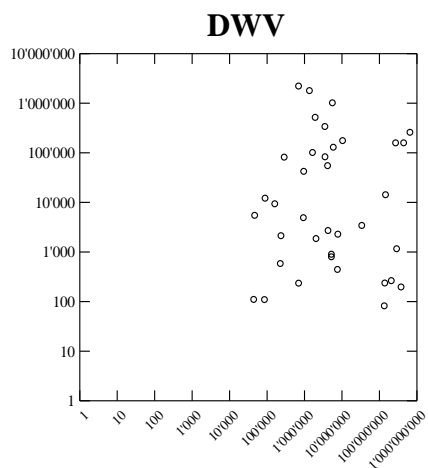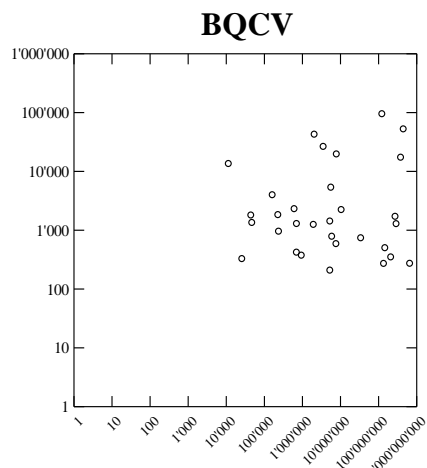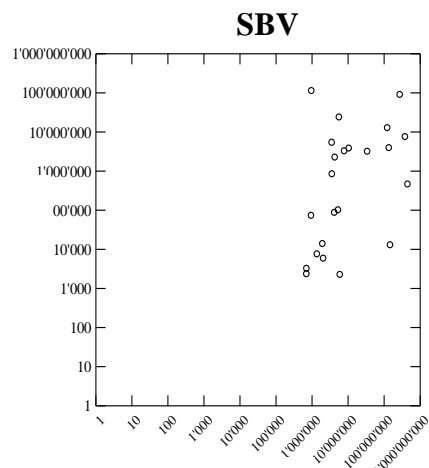

**Summer**

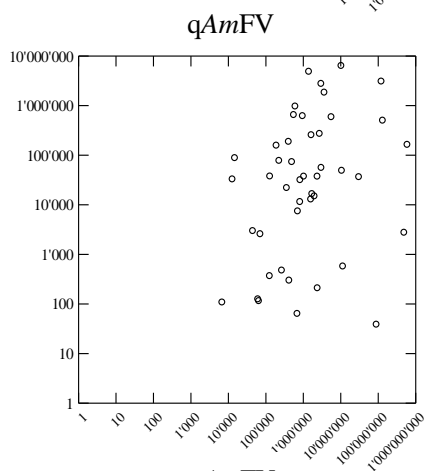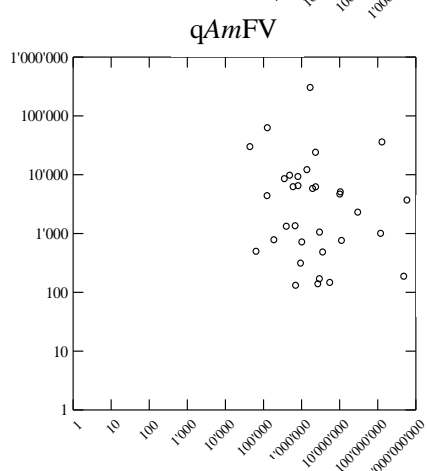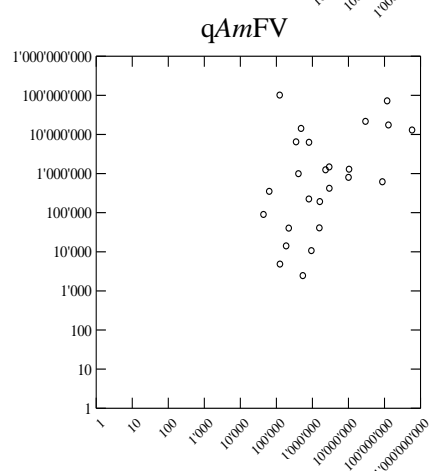

**Fall**

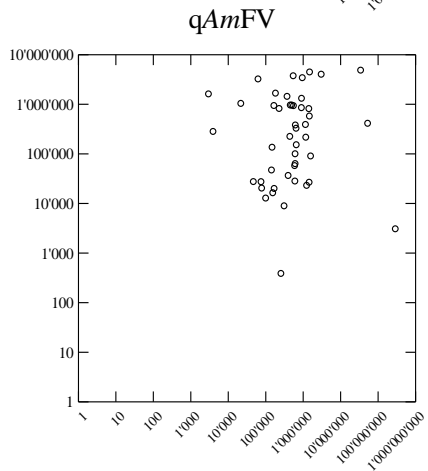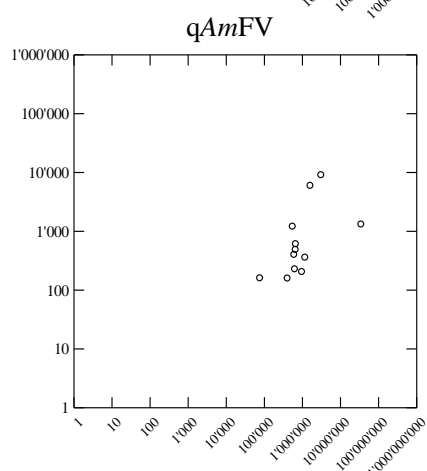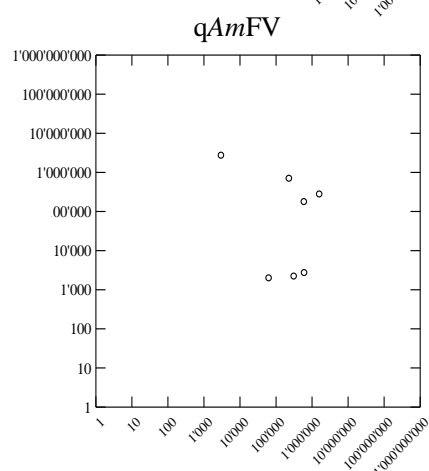

Supplement: Supplementary file 1 [file viruses-07-02654-s001.zip › FigureS1.pdf]
